# The United Nations Summit of the Future: An important opportunity for tobacco control

**DOI:** 10.18332/tid/183448

**Published:** 2024-02-03

**Authors:** Laurent Huber, Kelsey Romeo-Stuppy

**Affiliations:** 1 Action on Smoking and Health, Washington, United States

**Keywords:** United Nations, tobacco control, Summit of the Future, human rights

As reported in the Global Progress in the implementation of the WHO Framework Convention on Tobacco Control (FCTC)^1^, the second most often indicated barrier to implementation of tobacco control measures after tobacco industry interference, is the lack of or insufficient intersectoral cooperation and coordination. Fortunately, 2024 provides a mechanism we can all use to promote global governance cooperation and accelerate progress towards not only the WHO FCTC^2^ but also the UN Sustainable Development Goals (SDGs)^3^.

The United Nations Summit of the Future^4^ and its planned outcome, an action-oriented document called the Pact for the Future, has been framed as a once-in-a-generation opportunity to take cooperation within the United Nations to a new level. The Pact will be negotiated, and endorsed by countries in the lead-up to and during a Summit that will be held in September 2024. The UN describes the result as ‘a world – and an international system – that is better prepared to manage the challenges we face now and in the future, for the sake of all humanity and for future generations’. The process will be built on United Nations human rights treaties and environmental treaties as well as the SDGs, which does include the FCTC under Target 3.a which calls on countries to ‘strengthen the implementation of the World Health Organization Framework Convention on Tobacco Control’ and includes ‘age-standardized prevalence of current tobacco use among persons aged 15 years and older’ as an indicator^3^.

This process also explicitly builds on the final text of the outcome document called Addis Ababa Action Agenda adopted at the Third International Conference on Financing for Development held in 2015 and endorsed by the General Assembly in its resolution 69/313 of 27 July 2015^5^. The Addis Ababa Action Agenda which encourages multi-sectorial partnership and better coordination with global health initiatives, not only called on countries to strengthen implementation of the FCTC but also noted ‘that, as part of a comprehensive strategy of prevention and control, price and tax measures on tobacco can be an effective and important means to reduce tobacco consumption and healthcare costs, and represent a revenue stream for financing for development in many countries’^6^.

Young people and future generations have the right to grow up in a tobacco-free world; a concept, formalized by documents such as the Cape Town Declaration on Human Rights and a Tobacco Free World^7^, that has now become a reality as numerous jurisdictions have adopted tobacco-free future generation targets, such as the European Union, Canada, Denmark, England, France, Iceland, New Zealand, Norway, Scotland and Sweden, among others. A tobacco-free future will help protect the right to health of citizens of the world. Member States and other stakeholders must ensure that the huge potential of a tobacco-free world, across various human rights and sustainable development goals, is included in the Pact for the Future.

If the Summit of the Future is to live to its potential, the WHO FCTC needs to be fully integrated in this process along with other UN treaties. This means that countries will need to ensure that the WHO FCTC is mentioned along with other UN treaties listed in the Pact for the Future. On the other hand, countries need to remember that the number one obstacle to the implementation of the life saving measures in the FCTC is tobacco industry interference. For this reason, Member States and United Nations organizations must adhere to FCTC Article 5.3 – exclusion of the tobacco industry from public policy making. The tobacco industry is not a legitimate stakeholder and is an obstruction to the achievement of the human right to health, other human rights, and sustainable development.

## Data Availability

Data sharing is not applicable to this article as no new data were created.
